# Account of Diseases in an Eastern District of London, from December 20, 1803, to January 20, 1804

**Published:** 1804-02-01

**Authors:** 


### *Account of Diseases in an Eastern District of London, from December* 20, 1803, *to January* 20, 1804.

| | | | |
|---|---|---|---|
| ACUTE DISEASES. | | Amenorrhœa - - - - - | 14 |
| Peripneumonia Notha - | 4 | Leucorrhœa - - - - | 11 |
| Dysenteria - - - - - - | 2 | Menorrhagia - - - - | 6 |
| Variolæ - - - - - - | 2 | Hysteria - - - - - - | 3 |
| Rheumatismus Acutus - | 3 | Epilepsia - - - - - - | 1 |
| CHRONIC DISEASES. | | Cephalalgia - - - - - | 9 |
| Tussis - - - - - - | 14 | Vertigo - - - - - - - - | 4 |
| Tussis cum Dyspnœa - | 12 | Rheumatismus Chronicus | 17 |
| Catarrhus - - - - - - | 4 | PUERPERAL DISEASES. | |
| Phthisis Pulmonalis - - | 3 | Menorrhagia Lochialis - | 8 |
| Hydrothorax - - - - - | 2 | Dolores post Partum - | 7 |
| Anasarca - - - - - | 4 | Mastodynia - - - - - | 5 |
| Ascites - - - - - - - | 2 | Ephemera - - - - - - | 10 |
| Icterus - - - - - - - | 1 | INFANTILE | |

| | | | |
|---|---|---|---|
| INFANTILE DISEASES. | | Vermes | 5 |
| Convulsio | 3 | Herpes | 7 |
| Ophthalmia | 2 | Dentitio | 4 |

Since the last Report nothing has occurred particularly worthy of communication.

To the above list we may subjoin a general remark on the state of disease during the last year. In the earlier months of it, scarlatina made its appearance; it assumed however a mild form, and in very few instances proved fatal. In the spring, the influenza prevailed in so general a manner, and extended itself over so wide a circle as to occupy the principal attention of practitioners, not only in the metropolis, but in every part of the kingdom, and indeed in various foreign parts. So particular have been the reports communicated in this Journal, as well as in many distinct publications, that it is quite unnecessary to enter into detail of its symptoms or cure. After this disease had disappeared, an unusual degree of health was enjoyed for some months; the number of complaints was small, and their symptoms were uncommonly mild. The autumn also passed over without any remarkable interruption to the health of the public: the instances of cholera and dysentery, which in general are frequent at this season of the year, were very few. Since the winter commenced the temperature of the air has for the most part been so mild, that coughs, catarrhs, and the various pneumonic diseases that usually prevail at this season have been remarkably few.

